# Novel Pharmaceutical Strategy for Selective Abrogation of TSP1-Induced Vascular Dysfunction by Decoy Recombinant CD47 Soluble Receptor in Prophylaxis and Treatment Models

**DOI:** 10.3390/biomedicines9060642

**Published:** 2021-06-03

**Authors:** Molly Yao, Jalicia Sturdivant, Aren Ebrahimi, Samayita Ganguly, Tamer Elbayoumi

**Affiliations:** 1 Department of Pharmaceutical Sciences, College of Pharmacy-Glendale, Midwestern University, Cholla Hall 216, 19555 N. 59th Ave., Glendale, AZ 85308, USA; sgangu@midwestern.edu; 2 College of Graduate Studies, Midwestern University, Science Hall, 19555 N. 59th Ave., Glendale, AZ 85308, USA; 3 Arizona College of Osteopathic Medicine, Midwestern University, Glendale Hall, 19555 N. 59th Ave., Glendale, AZ 85308, USA; jalicia.sturdivant@icloud.com (J.S.); ebrahimiaren@gmail.com (A.E.)

**Keywords:** thrombospondin 1, CD47, decoy receptor protein, vasorelaxation, endothelium, pulmonary arterial hypertension, ischemia/reperfusion injury

## Abstract

Elevated thrombospondin 1 (TSP1) is a prevalent factor, via cognate receptor CD47, in the pathogenesis of cardiovascular conditions, including ischemia-reperfusion injury (IRI) and pulmonary arterial hypertension (PAH). Moreover, TSP1/CD47 interaction has been found to be associated with platelet hyperaggregability and impaired nitric oxide response, exacerbating progression in IRI and PAH. Pathological TSP1 in circulation arises as a target of our novel therapeutic approach. Our “proof-of-concept” pharmacological strategy relies on recombinant human CD47 peptide (rh-CD47p) as a decoy receptor protein (DRP) to specifically bind TSP1 and neutralize TSP1-impaired vasorelaxation, strongly implicated in IRI and PAH. The binding of rh-CD47p and TSP1 was first verified as the primary mechanism via Western blotting and further quantified with modified ELISA, which also revealed a linear molar dose-dependent interaction. Ex vivo, pretreatment protocol with rh-CD47p (rh-CD47p added prior to TSP1 incubation) demonstrated a prophylactic effect against TSP1-impairment of endothelium-dependent vasodilation. Post-treatment set-up (TSP1 incubation prior to rh-CD47p addition), mimicking pre-existing excessive TSP1 in PAH, reversed TSP1-inhibited vasodilation back to control level. Dose titration identified an effective molar dose range (approx. ≥1:3 of tTSP1:rh-CD47p) for prevention of/recovery from TSP1-induced vascular dysfunction. Our results indicate the great potential for proposed novel decoy rh-CD47p-therapy to abrogate TSP1-associated cardiovascular complications, such as PAH.

## 1. Introduction

Under physiologic circumstances, homotrimeric glycoprotein thrombospondin 1 (TSP1) is ubiquitously expressed in extracellular matrix and stored in the α-granules of platelets, remaining very low in local sites and circulation [1]. Substantial elevation in circulatory TSP1—up to a thousand-fold higher—has been recognized as a biomarker in multiple pathologic conditions, including coronary artery disease (CAD), pulmonary arterial hypertension (PAH), type II diabetes, and cardiac allografts [2,3,4]. Other than a risk indicator, excess plasma TSP1 has been heavily involved in pathologic modulation and progress of these conditions [5,6]. In PAH patients and diseased murine models, overexpressed TSP1 has been shown to promote oxidative stress in lung tissues and to contribute towards simultaneous vascular dysfunction and subsequent vasculopathy [7,8]. Furthermore, upregulated TSP1 was not only revealed to crosslink cardiac and renal fibrosis in obesity and metabolic dysfunction but also correlated with insulin resistance as well as adipose inflammation in nondiabetic subjects.

The multifaceted TSP1-mediated actions, as exemplified above, rely on its potential to interact with a wide variety of molecules—based on domain mapping—such as cell-surface-bound receptors CD47 and CD36, growth factors and regulators vascular endothelial growth factor (VEGF) and platelet derived growth factor (PDGF), cytokine transforming growth factor (TGF), proteases thrombin and matrix metalloproteinase-2 (MMP2), and many more [9]. The mechanism underlying hypertension, PAH, and renal ischemia-reperfusion injury (IRI) depends on TSP1 binding to its cognate receptor CD47, which is typically expressed on the endothelial cell surface. This significantly reduces the bioavailability of endogenous and exogenous nitric oxide (NO) by either inhibiting the activity of endothelial nitric oxide synthase (eNOS) and soluble guanylate cyclase [10,11,12] or inducing ROS generation by vascular smooth muscle cells [13]. TSP1/CD47 ligation is also indicated in left ventricular heart failure in a distinct Ca^2+^/calmodulin protein kinase II (CaMKII)-dependent manner [14]. The versatile interactions of TSP1 with CD47 and CD36 on endothelial cells have been established to promote thrombosis and inhibit angiogenesis, respectively [15,16,17].

Cluster of differentiation protein 47 (CD47) or integrin associated protein (IAP) is a highly glycosylated plasma membrane protein expressed in different cell types of all tissues. In addition to the effects initiated by direct binding with TSP1, CD47 is recognized as a “self” marker on the cell surface, displaying a “do not eat me” signal to immune cells via interaction with signal regulatory protein α (SIRPα), thus rendering cells rich in CD47 expression immune to phagocytosis [18,19]. Some strategies interfering with phagocyte-modulation by CD47—a few related to its putative 4N1K interaction domain on TSP1—have been explored [20]. Immunotherapy specifically interrupting CD47/SIRPα communication, namely, anti-CD47 monoclonal antibody Hu5F9-G4, is currently under six clinical trials and has emerged as a breakthrough in cancer treatment for solid and hematological tumors [21]. Analogously, human anti-TSP1 peptides and monoclonal antibodies have been developed experimentally but never into functional clinical strategy. This is possibly due to the pluripotent interactions of TSP1, with many biological molecules within cardiovascular and immune systems [20,22]. To date, there is no specific intervention to prevent or overcome the detrimental effects caused by excessive TSP1 in circulation by precisely targeting the endothelial TSP1/CD47 axis, mainly implicated in several critical cardiovascular pathologies.

Decoy receptor protein (DRP) is a unique and established method of signal inhibition that is repeatedly employed in the field of immunology; it has been defined as “a receptor that is able to recognize and bind specific growth factors or cytokines efficiently but is not structurally able to signal or activate the intended receptor complex” [23,24]. Such an immunological signal inhibition approach has successfully evolved into a therapeutic design, exemplified through clinical drug Enbrel^®^ (etanercept), currently approved for the treatment of rheumatoid arthritis, psoriatic arthritis, plaque psoriasis, and ankylosing spondylitis [25]. Etanercept is a biopharmaceutical recombinant fusion protein of TNF receptor-2 and the Fc portion of IgG_1_ that functions as a soluble DRP, binding its ligand TNF-α and keeping it from binding to its regular cell-surface-expressed receptor on white blood cells [25]. Likewise, Prolia^®^ (denosumab) has been prescribed to inhibit bone resorption occurring in osteoporosis, bone cancer, and bone metastasis secondary to other cancers via acting as a DRP for RANKL [26].

Here, we propose a truncated CD47 protein, constituting the domain known to only bind to TSP1, to serve as a prospective DRP candidate, which offers three therapeutic advantages. First, our DRP—exogenous soluble recombinant human CD47 peptide (rh-CD47p) containing a TSP1-binding sequence in two putative interaction regions—can compete with the endogenous CD47 protein expressed on endothelial cell membranes for the CD47-binding groove in TSP1, typically involving the 4N1K hydrophobic cleft RFYVVMWK peptide region on the TSP1 C-terminal domain [22,27], to intercept the pairing up of plasma TSP1 and endothelial CD47. Subsequently, the soluble decoy rh-CD47p will block the TSP1/CD47-mediated endothelial effects. Second, our DRP rh-CD47p, by actively binding and scavenging excess TSP1 molecules in circulation, will result in the formation of scavenging complexes of rh-CD47p/TSP1, which will ultimately be eliminated from the body via the mononuclear phagocyte system (MPS). Thus, MPS elimination of the rh-CD47p/TSP1 scavenging complex can effectively reduce the pathologically elevated TSP1 in circulation back to normal or possibly subnormal levels. Lastly, the hypothesized DRP strategy can be further enhanced through pharmaceutical modification of DRP, e.g., covalently grafting multiple DRP (rh-CD47p) molecules onto the surface of nanocarriers, thus increasing the number of active DRP-molecules as nanomedicine for pharmaceutical application.

The current “proof-of-concept” report of our DRP, rh-CD47p, has carefully examined its pharmacological effects in a dose-dependent approach against target protein TSP1 and revealed the underlying mechanism. Association between the ectodomains of CD47 and TSP1 was validated in different laboratories [28,29], while a direct insight into TSP1/CD47 ligation was acquired through a simulation study rather than a classic approach of X-ray crystallography [27]. Hence, our pharmacological data, here, is also complemented by in silico data, further elucidating the putative details of the molecular interaction between the extracellular domain of CD47 [27], represented in our soluble DRP (rh-CD47p) and target plasma protein, TSP1.

## 2. Materials and Methods

### 2.1. In Silico Modeling and Analysis

#### 2.1.1. Simulations

Molecular dynamics simulations and normal mode analysis of the TSP1 C-terminal domain were performed using the PDB:1UX6 crystallographic structure [30] as a starting point (1.9 Å resolution). A constant temperature and pressure (300 K; 1 atm.) MD simulation of the protein, immersed in a water box, was performed with Molecular Operating Environment (MOE) using the CHARMM force field [31,32]. An integration time-step of 1 fs was used, the bonds containing a hydrogen atom being held constant with the SHAKE algorithm. Electrostatic interactions were treated with the Particle Mesh Ewald (PME) method [27,33]. A switching function between 8 and 10 Å was used for van der Waals interactions. The Vega ZZ program was initially used to translate model PDB (Protein Data Bank) files containing detailed molecular structure information into a picture [34,35].

#### 2.1.2. Energy Minimization and Calculating the Active Site Sequence

The energy of the protein molecule was minimized using the Energy Minimization algorithm of the MOE tool. The following parameters were used for energy minimization: gradient: 0.05, Force Field: MMFF94X+Solvation, Chiral Constraint: Current Geometry. Energy minimization was terminated when the root mean square gradient fell below 0.05. The minimized TSP1 structure was used as the template for Docking [27,36].

Active sites present in the TSP-1 protein were identified from the 3D atomic coordinates of the receptor using Q-SITE FINDER. It is an energy-based method for the prediction of protein-ligand binding sites [35].

#### 2.1.3. Building of the rh-CD47p Model and Protein–Protein Docking Experiments

For the preparation of each protein molecule, the protein sequence was loaded to the sequence editor tool, and it was searched for a template. The entire extracellular domain of human CD47 was identified as the V set domains of the immunoglobulin-like superfamily [27]. The template 2CMZ was selected for the preparation of the homology model. Based on earlier reports for the identified fold (Z score = 17.37), the structure of the extracellular domain (PDB: 2ICC) was chosen as a template [27]. The binding of this model was selected to realize protein–protein docking experiments toward the low-energy conformation of TSP1, using the MOE docking program to find the correct conformation and configuration of the ligand to obtain minimum energy structure. The proposed parameters for Docking were Total Runs = 100, Cycle/Runs = 20, Iteration Limit = 10,000, Potential Energy Grid: ON, Annealing Algorithm: Simulated Annealing [31].

### 2.2. Materials and Reagents

The drugs used were obtained from the following sources: L-phenylephrine hydrochloride, acetylcholine chloride (Sigma-Aldrich, St. Louis, MO, USA), native human thrombospondin 1 (Novus Biologicals, Littleton, CO, USA; Athens Research & Technology, Athens, GA, USA; Thermo Fisher Scientific, Anthem, AZ, USA), recombinant human thrombospondin 1 (R&D Systems, Minneapolis, MN, USA; BioVision, Milpitas, CA, USA), recombinant human CD47 peptide (Bon Opus, previously NovoProtein, Millburn, NJ; Abcam, Cambridge, MA, USA). All drugs were dissolved and diluted in physiological salt solution. All animal studies were performed under a protocol approved (16 August 2018) by the Midwestern University Institutional Animal Care and Use Committee (IACUC) in accordance with NIH guidelines. Male C57BL/6 mice were obtained from the Jackson Laboratory (Bar Harbor, ME, USA).

### 2.3. Methods

#### 2.3.1. Measurement of Mouse Thoracic Aorta Relaxation

Male C57BL/6 mice (6–8 weeks) were first anesthetized with isoflurane inhale. Following trans-section of the abdominal vena cava, the vasculature was gently flushed with cold 0.9% saline via puncture of the left ventricle to remove blood. Thoracic aortas were dissected free, cleared of surrounding adherent adipose tissue, and placed in standard Krebs buffer (composition in mM: NaCl, 118.4; KCl, 4.7; CaCl_2_, 1.6; MgSO_4_, 1.2; KH_2_PO_4_, 1.2; NaHCO_3_, 14.9; dextrose, 5.5; Na_2_Ca EDTA, 0.026; meclofenamic acid, 0.0031; pH 7.4). Aorta segments of 3 mm length were mounted on a multiwire myograph (Danish Myo Technology, Atlanta, GA, USA) in 5 mL Krebs buffer maintained at 37 °C, gassed with 95% O_2_/5% CO_2_, and brought to an optimal resting tension of 500 mg. Viability of the vessels was ascertained by a contractile response to potassium chloride (60 mM) in Krebs buffer. Endothelium intactness was assessed on vascular tone change in aortic rings contracted by phenylephrine (1 μM), followed by a single concentration of acetylcholine (1 μM). Only aortic rings demonstrating 80% of relaxation or greater were considered endothelium-intact and selected for further experimentation. In precontracted vessels with a single concentration of phenylephrine (1 μM), accumulative endothelium-dependent vasodilation to acetylcholine (1 nM to 10 μM) in semilog increments was then tested with or without the indicated treatments detailed below, along with the appropriate controls [11].

#### 2.3.2. Protocol for Pretreatment with Recombinant Human CD47 Peptide (rh-CD47p)

Myograph-mounted aortic segments received rh-CD47p treatment 15 min prior to the addition of TSP1. Total incubation time for TSP1 was maintained for 60 min, with rh-CD47p for 75 min (*n* = 3–6) [11]. Doses of rh-CD47p are expressed in molar ratios of TSP1 to rh-CD47p.

#### 2.3.3. Protocol for Post-Treatment with rh-CD47p

Post-treatment protocol reversed the sequence of addition of rh-CD47p and TSP1 and maintained the same incubation time for TSP1. Initially, TSP1 was incubated for 15 min, then rh-CD47p was added for an incubation period of 45 min. Total treatment time for TSP1 was kept for 60 min (*n* = 3–4) [11,37]. Doses of rh-CD47p are expressed in molar ratios of TSP1 to rh-CD47p.

#### 2.3.4. Immunoblot

A mixture of TSP1 and rh-CD47p, with or without the monoclonal TSP1 antibody (mAb 301221, Novus Biologicals, Littleton, CO, USA), was prepared in a cell-free setting and incubated at 37 °C for 45–60 min. Upon incubation, TSP1 and rh-CD47p mixture samples were subject to electrophoresis on gradient 4–20% Mini-PROTEAN TGX precast gels (BioRad, Hercules, CA, USA) for approximately 1 h at 200 V, followed by transfer to a nitrocellulose membrane (BioRad, Hercules, CA, USA) at 4 °C with a constant current of 150 mA for 2 h. Membranes treated with Odyssey blocking buffer (LI-COR, Lincoln, NE, USA) at room temperature for 1 h were exposed to a primary antibody against TSP1 (A6.1, Abcam, Cambridge, MA, USA, 1:500) at 4 °C overnight and subsequent incubation with a red-fluorescence-labeled secondary antibody (926-68050, LI-COR, Biosciences, Lincoln, NE, USA, 1:10,000) at room temperature for 1 h. Membranes were reprobed with a primary antibody against rh-CD47p (EPR21794, Abcam, Cambridge, MA, USA, 1:500) at 4 °C overnight and a green-fluorescence-labeled secondary antibody (926-32213, LI-COR, 1:10,000) at room temperature for 1 h. Images were captured by an Odyssey CLx imagining system (LI-COR, Biosciences, Lincoln, NE, USA).

#### 2.3.5. Modified CD47 ELISA

Although CD47 ELISA detects the CD47 peptide in a mixture, it does not distinguish between peptides complexed with TSP1 and those remaining unbound/free, and, thus, a separation process of mixture samples prior to CD47 ELISA was necessary to facilitate the determination of a stoichiometric relation between rh-CD47p and TSP1. TSP1 was mixed with a fixed amount of rh-CD47p in increasing molar ratios, starting from 1:1 up to 1:9 in cell-free settings, then incubated at 37 °C for 45–60 min. The mixtures were then filtered through Amicon Ultra centrifugal filter devices (Millipore Sigma, Billerica, MA, USA) following the manufacturer’s user guide. Briefly, Amicon Ultra 0.5 mL centrifugal filter devices in a nominal molecular weight limit of 100 K were loaded with the same volume of rh-CD47p control or mixtures of TSP1 and rh-CD47p and centrifuged at 12000× *g* for 30 min at 4 °C. Finally, the levels of rh-CD47p in the collected filtrates were quantified via a DuoSet^®^ ELISA kit for human CD47 (R&D Systems, Minneapolis, MN, USA) per the manufacturer’s instructions (*n* = 5–7). In brief, a capture antibody-coated 96-well microplate was maintained at room temperature overnight. Following blocking with reagent diluent for a minimum of 1 h at room temperature, the filtrates, prepared in 2-fold serial dilution with reagent diluent, were loaded onto the plate and incubated at room temperature for 2 h. Subsequent detection antibody incubation was kept at room temperature for 2 h. A 20-min incubation with streptavidin-HRP, followed by another 20-min with substrate solution, was kept at room temperature with protection from light. The optical density (OD) was measured spectrophotometrically at 450  and 560 nm using a microplate reader (multidetector microplate reader VICTOR™ X3, PerkinElmer, Shelton, CT). The concentration of rh-CD47p in the filtrates extracted from rh-CD47p control and the mixtures of TSP1 and CD47 was determined against the standard calibration curve of the R&D CD47 standard.

### 2.4. Data Analysis

Data were reported as mean ± standard error mean (SEM) of a minimum of three independent experiments. Cumulative concentration-response curves were constructed by increasing the concentration cumulatively in half-log increments until a maximal response was obtained. Data analysis and plotting of concentration-response curves using nonlinear regression fitting were performed with Graphpad Prism 8.0.0 (GraphPad Software, San Diego, CA, USA). Comparisons of concentration-response curves between the groups were performed using two-way ANOVA followed by a Bonferroni posthoc test. The logarithms of EC50 values were compared using a two-tailed Student’s *t*-test. Comparisons of unbound rh-CD47p, isolated from mixtures with TSP1, were carried out using one-way ANOVA followed by a Bonferroni posthoc test. A *p*-value < 0.05 was taken as significant.

## 3. Results

### 3.1. In Silico Modeling and Visualization of Putative Extracellular CD47:TSP1 Interactions

**Figure 1:**
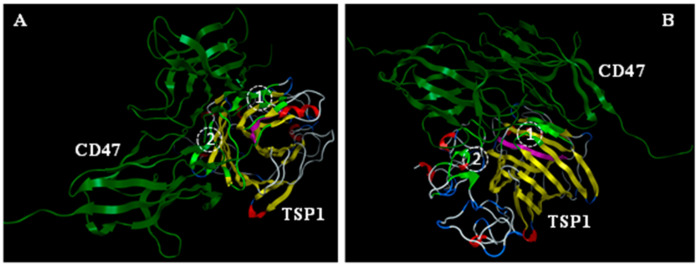
Molecular simulation of ligand, TSP1, interaction with the receptor (CD47) in a solvent-free environment. Slightly open conformation of the 4N1K hydrophobic cleft (RFYVVMWK peptide region, pink) on TSP1 C-terminal domain was modeled via Molecular Operating Environment (MOE) to participate in interaction with the N-terminus of the model of the soluble extracellular part of the CD47 receptor (shown in two mode views, (**A**,**B**), as identified experimentally, in addition to the two putative TSP1:CD47 interaction regions (1) and (2), respectively, as recognized earlier. Light green: residues of TSP1 involved in binding.

Consistent with an earlier molecular modeling report on a solvent-free environment [22,27], computational modeling of TSP1 interactions with model recombinant human CD47 peptide (rh-CD47p)—based on a homology model of the extracellular part of the CD47 receptor—evoked an open conformation of the TSP1 C-terminal domain for protein-protein docking experiments (Figure 1). Similarly, the slightly open conformation of the 4N1K hydrophobic cleft RFYVVMWK peptide region on the TSP1 C-terminal domain seemed to also participate, but not exclusively, in interactions with the N-terminus of model rh-CD47p [22,38], revealing two putative CD47:TSP1 interaction regions, (1) and (2), as shown in two example simulation modes (Figure 1A,B).

### 3.2. In Vitro Confirmation of Qualitative and Quantitative Association between mTSP1 and Soluble rh-CD47p

**Figure 2:**
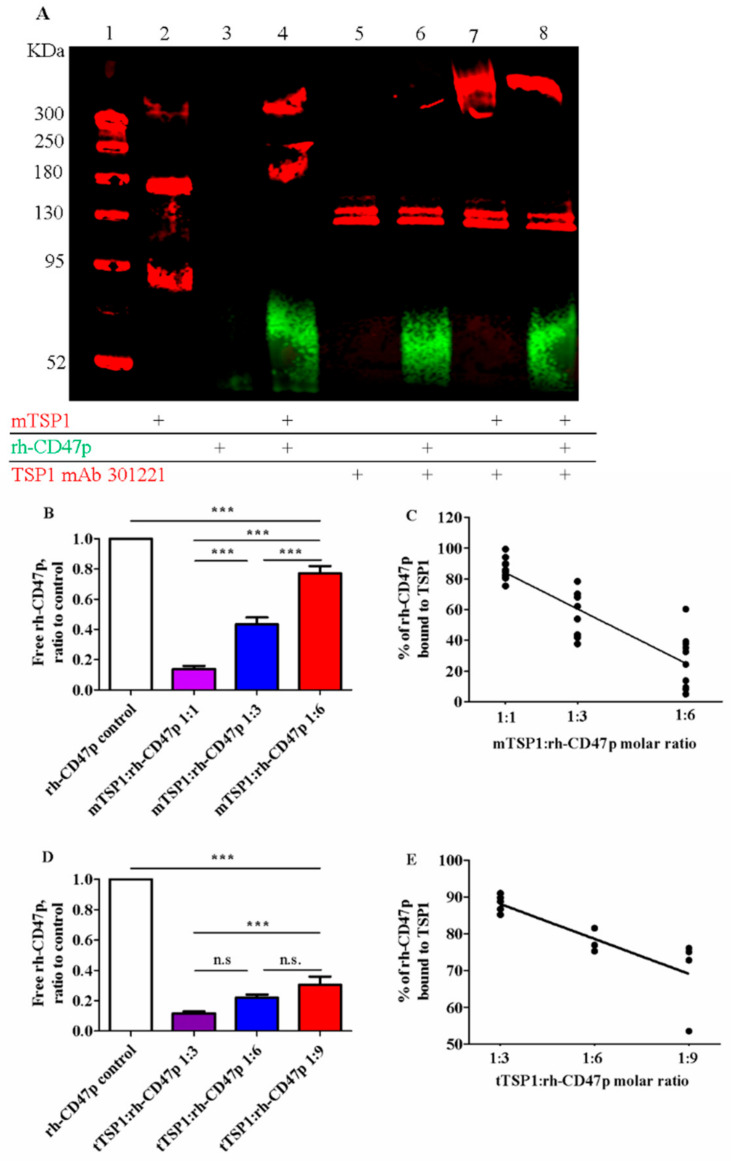
Qualitative and quantitative confirmation of association between TSP1 and the recombinant human CD47 peptide (rh-CD47p). (**A**) Representative image of immunoblots of combination of monomer TSP1 (mTSP1) and rh-CD47p incubated at 37 °C for 60 min in a cell-free setting. Lane 1: molecular weight marker; 2: mTSP1 control; 3: rh-CD47p control; 4: mTSP1+rh-CD47p mixture; 5: TSP1 blocking antibody alone (TSP1 mAb 301221); 6. rh-CD47p+TSP1 blocking antibody; 7: mTSP1+TSP1 blocking antibody; and 8: mTSP1+rh-CD47p mixture with TSP1 blocking antibody. mTSP1 control (Lane 2) is present as a clear band in red fluorescence at 180 KDa; rh-CD47p control (Lane 3) is presented as a smear and a widely spread band in green fluorescence at approximately 60 KDa due to glycosylation; the mixture of mTSP1 and rh-CD47p without TSP1 blocking antibody at 240 KDa (Lane 4) or stacked up greatly higher than 300 KDa, following treatment with TSP1 blocking antibody (Lane 8), are presented in red fluorescence. Quantitative determination of binding of rh-CD47p to mTSP1 was performed via modified ELISA. A fixed amount of rh-CD47p was co-incubated with mTSP1 or trimeric TSP1 (tTSP1) at 37 °C for 60 min in a cell-free setting. Unbound rh-CD47p was collected and subject to human CD47 ELISA. Free rh-CD47p, normalized to control, increased as the amount of mTSP1 (**B**) or tTSP1 (**D**) decreased in the mixtures (one-way ANOVA followed by a Bonferroni posthoc test; Panel B: *n* = 7, *** *p* < 0.0001, mTSP1:rh-CD47p 1:1 to 1:6 vs. rh-CD47p control, mTSP1:rh-CD47p 1:1 vs. 1:3 and 1:6, and mTSP1:rh-CD47p 1:3 vs. 1:6; Panel D, *n* = 5, *** *p* < 0.001, tTSP1:rh-CD47p 1:3 to 1:9 vs. rh-CD47p control, tTSP1:rh-CD47p 1:3 vs. 1:9; n.s., no significance, tTSP1:rh-CD47p 1:3 vs. 1:6 and 1:6 vs. 1:9). Correlation between DRP doses, expressed in a molar ratio of TSP1 to rh-CD47p, and the bound rate of DRP was constructed for combination with mTSP1 (**C**) and tTSP1 (**E**), respectively (Panel C, *p* < 0.0001; Panel E, *p* < 0.01).

It has been well known that TSP1 binds to its cognate receptor CD47 via the C-terminal RFYVVM motif. Our in silico data, corroborated by earlier simulation reports [27], also indicate such recognized TSP1-interaction with the soluble CD47 extracellular domain-derived protein, rh-CD47p, constituting our DRP molecule. Therefore, DRP administration is proposed to block TSP1-induced detrimental vascular effects via direct association with target recombinant human TSP1, identical to a single subunit of the endogenous trimeric protein product. In vitro, the binding of rh-CD47p to TSP1 was first verified via immunoblotting, in which the functionally smaller template, the monomeric form of TSP1 (mTSP1), was simply coincubated with DRP in a cell-free setting to avoid a complicated protein reduction of the larger trimeric TSP1, tTSP1. Following monoclonal TSP1 antibody detection (clone A6.1), a marginally smeared band—corresponding to the simple mixture of mTSP1 and rh-CD47p—appeared at 240 KDa, showing an upward shift from the sharp band of mTSP1 alone at 180 KDa (Figure 2A). The extra 60 KDa increase, detected in an analyzed admixture sample (3:1 molar ratio mixture of mTSP1 and rh-CD47p), unambiguously confirmed the direct mTSP1 association with glycosylated rh-CD47p in such exclusive scenario, which was further verified after including a blocking antibody against mTSP1 (mAb 301221) in the samples. In this situation, Lanes 5-8 clearly show that the mTSP1 blocking antibody did not associate with rh-CD47p (Lanes 5–6), while mTSP1 only and mTPS1 in admixture stacked at a higher molecular weight than 300 KDa (detected here via a different monoclonal TSP1 antibody, A6.1, see Lanes 7–8) due to aggregation with the mTSP1 blocking antibody (Figure 2A).

Quantitative analysis of the dosimetric association between DRP molecules and TSP1 was investigated via modified human CD47 ELISA. Gradually decreasing mTSP1 content in the mixtures, from 1:1, 1:3, down to 1:6, as molar ratios with rh-CD47p was associated with an increase in free rh-CD47p, from 13.6 ± 2.2%, 43.4 ± 4.6% to 77.1 ± 4.6%, respectively (Figure 2B). Across the tested TSP1:DRP molar ratio dose range, a linear correlation between the rates of DRP bound to target protein mTSP1 and the DRP doses was constructed, with a correlation coefficient of 0.83 (Figure 2C). This modified ELISA was then repeated with the physiological trimeric form of TSP1 (tTSP1), considering the potential impact of its complex multisubunit structure. In a similar pattern, increasing fractions of unbound DRP were collected from the tTSP1:rh-CD47p mixture samples, from 11.7 ± 1.0%, 22.1 ± 1.8% to 32.9 ± 6.8%, as tTSP1 molar concentration ratios in these mixtures decreased from 1:3, 1:6, down to 1:9, respectively (Figure 2D). Analogously, rh-CD47p doses were also found to correlate with the tTSP1-bound rates (calculated correlation coefficient r = 0.81) (Figure 2E).

Together, our qualitative and quantitative in vitro study results confirmed a direct binding mechanism between soluble CD47 peptide and TSP1, as proposed by the in silico data, which was shown to behave in a virtually linear concentration-dependent manner.

### 3.3. Ex Vivo Inhibition of Endothelium-Dependent Vasodilation Due to Trimeric and Monomeric Forms of TSP1

**Figure 3:**
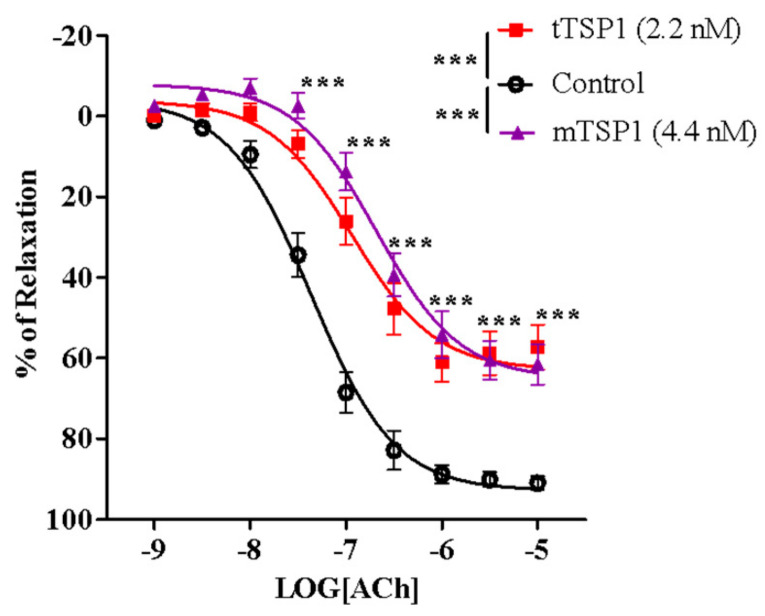
Trimeric TSP1 (tTSP1) at 2.2 nM and monomeric TSP1 (mTSP1) at 4.4 nM showed significant inhibition on vasodilation to the same extent (two-way ANOVA followed by a Bonferroni posthoc test; *n* = 6, *** *p* < 0.0001, mTSP1 or tTSP1 vs. control; *p* > 0.05, mTSP1 vs. tTSP1). TSP1, in both conformations, inhibited endothelium-dependent vasodilation stimulated by acetylcholine, from 30 nM to 10 μM (*n* = 6, *** *p* < 0.0001, mTSP1 or tTSP1 vs. control; *p* > 0.05, mTSP1 vs. tTSP1).

Since it was first discovered, TSP1 has been identified as a homotrimeric glycoprotein with a molecular weight between 420 and 450 KDa. The three-subunit structure of this large protein may interfere with the conformational change of individual branches when exposed to membrane-bound CD47, potentially complicating some experimental situations when studying TSP1 interactions with our DRP, rh-CD47p. Hence, a recombinant human TSP1 monomer, mTSP, comprising the entire amino acid sequence of a single subunit, was also studied here. It was included as a practical smaller template of the endogenous trimeric target protein, tTSP1, which is typically extracted and purified from human platelets. Initially, in our ex vivo model, the efficacy of the mTSP1 substitute was evaluated against that of its native trimeric form. Several doses of mTSP1 were tested, 2.2–8.8 nM vs. an often-used pathologically relevant dose of tTSP1 at 2.2 nM (1 μg/mL), owing to its estimated native triple activity. Only 4.4 nM of mTSP1 induced inhibition of acetylcholine-stimulated vasodilation equivalent to that induced by 2.2 nM of tTSP1 (Figure 3), while higher concentrations of mTSP1 (≥ 6.6 nM) did not induce a noticeable further impairment of vasodilation (data not shown), indicating plateau levels were reached. Overall, EC_50_ of acetylcholine in the presence of mTSP1 or tTSP1 was rightward shifted by 5.5- or 4.9-fold, respectively (Table 1), and mTSP1 proportionately replicated tTSP1-mediated vasodilation impairment across a 1000-fold change in concentrations of acetylcholine, as expected, showing significant reduction in vasodilation vs. vehicle control (*p* < 0.001). Consequently, mTSP1 at 4.4 nM was consistently applied for all subsequent studies, in parallel with tTSP1 at 2.2 nM.

### 3.4. Pretreatment with rh-CD47p Prevents TSP1-Blunted Vasodilation

We hypothesized that the administration of soluble exogenous DRP containing a TSP1-binding sequence would interrupt communication between the elevated TSP1 in circulation and its membrane cognate receptor CD47, resulting in protection of the vascular bed against damages mediated by TSP1. Henceforth, in an initial experimental prophylaxis scenario, isolated vessels first received various doses of rh-CD47p 15 min prior to coincubation with TSP1 (in either monomeric or trimeric form) for 60 min—noted as pretreatment in this study.

**Figure 4:**
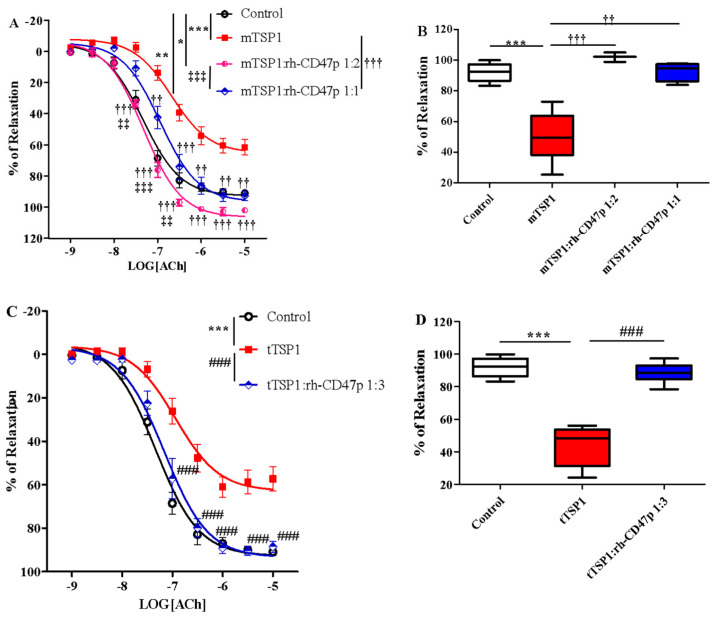
Pretreatment with recombinant human CD47 peptide (rh-CD47p) effectively prevents TSP1-blunted vasodilation. rh-CD47p was administered 15 min prior to incubation of TSP1. (**A**) Concentration-response curves of isolated mouse thoracic aorta exposed to monomeric TSP1 (mTSP1) alone or combinations of mTSP1 and rh-CD47p in two molar ratios. mTSP1-induced impairment of vasodilation was prevented via pretreatment with rh-CD47p (two-way ANOVA followed by a Bonferroni posthoc test; *n* = 3–6, *** *p* < 0.0001, mTSP1 vs. control; * *p* < 0.05, mTSP1:rh-CD47p 1:2 vs. control; ** *p* < 0.01, mTSP1:rh-CD47p 1:1 vs. control; †† *p* < 0.01 and ††† *p* < 0.001, mTSP1:rh-CD47p 1:1 or 1:2 vs. mTSP1; ‡‡ *p* < 0.01, and ‡‡‡ *p* < 0.001, mTSP1:rh-CD47p 1:1 vs. 1:2). (**B**) Percentages of relaxation for all treatment groups at 10 μM acetylcholine are presented as box and whisker plots showing median, 1st quartile, 3rd quartile, and 95% CI. Significant difference in relaxation was found between groups treated with mTSP1 alone and the combination of mTSP1 and rh-CD47p (*n* = 3–6, *** *p* < 0.001, mTSP1 vs. control; †† *p* < 0.01, mTSP1:rh-CD47p 1:1 vs. mTSP1; ††† *p* < 0.001, mTSP:rh-CD47p 1:2 vs. mTSP1). (**C**) Concentration-response curves of isolated mouse thoracic aorta exposed to trimeric TSP1 (tTSP1) alone or the combination of tTSP1 and rh-CD47p in a single molar ratio. tTSP1-induced impairment of vasodilation was completely blocked via pretreatment with rh-CD47p (two-way ANOVA followed by a Bonferroni posthoc test; *n* = 4, *** *p* < 0.0001, TSP1 vs. control; ### *p* < 0.0001, TSP1:rh-CD47p 1:3 vs. TSP1; *p* > 0.05, TSP1:rh-CD47p 1:3 vs. control). (**D**) Percentages of relaxation for all treatment groups at 10 μM acetylcholine are presented as box and whisker plots showing median, 1st quartile, 3rd quartile, and 95% CI. rh-CD47p treatment protected the vessel from substantial inhibition of vasodilation by native TSP1 (*n* = 4, *** *p* < 0.0001, tTSP1 vs. control; ### *p* < 0.0001, tTSP1:rh-CD47p 1:3 vs. tTSP1).

The prophylactic effect of rh-CD47p against TSP1-impaired vasodilation was first investigated using two different dose levels of rh-CD47p. Both rh-CD47p pretreatment levels effectively neutralized mTSP1-exacerbated vasoconstriction, demonstrating dose-dependent increased potency and augmented vasorelaxation (Figure 4A). Quantitatively, increasing the molar ratio dose of rh-CD47p pretreatment from 1:1 to 1:2 resensitized mTSP1-treated vessels to acetylcholine by 2- and 5-fold, respectively, compared to the mTSP1-treated group (Table 1). Moreover, vascular tone prophylaxis—as imparted by both doses of rh-CD47p pretreatment—against mTSP1 inhibition was evident through marked improvement in acetylcholine-induced vasodilation (starting at 30 nM to 10 μM). Indeed, while the mTSP1 challenge reduced the vasorelaxation induced by 10 μM acetylcholine from 92.0 ± 1.4% in vehicle control down to 61.6 ± 5.0%, pretreatment with rh-CD47p retained vasorelaxation at 92.7 ± 3.1% and 102.0 ± 1.7% when applied at dose ratios 1:1 and 1:2, respectively (Figure 4B). Furthermore, a significant difference in counteracting TSP1 inhibition was noted between both rh-CD47p doses, in which doubling the molar ratio of DRP to mTSP1 achieved complete abrogation of mTSP1-mediated inhibition of vasorelaxation. At the same time, equimolar concentrations of rh-CD47p to mTSP1 rendered significant partial prevention of vascular tone deterioration (Figure 4A). In addition to distinct efficacy in improving sensitivity to acetylcholine-induced vasodilation, a slightly higher molar dose of DRP (namely, a 1:2 mTSP1:rh-CD47 ratio) resulted in markedly greater vasorelaxation in response to the entire mid-range of acetylcholine concentrations between 30 and 300 nM (Figure 4A, *p* < 0.01, <0.001, mTSP1:rh-CD47p 1:1 vs. mTSP1:rh-CD47p 1:2).

Effective prophylaxis provided by rh-CD47p was also successfully reproduced in the same vascular functional assay in the presence of tTSP1 (native polymeric conformation), evident through yielding almost identical concentration-response curves as the vehicle-only controls (Figure 4C). Specifically, the vascular tone induced by acetylcholine was improved very close to control (Table 1); meanwhile, vasorelaxation stimulated by mid- to high-range acetylcholine (100 nM to 10 μM) was well preserved in the case of pretreatment with rh-CD47p. For instance, vasorelaxation induced by the highest concentration of acetylcholine (10 μM) improved from 48.6 ± 6.8% in the presence of tTSP1, up to 88.6 ± 2.5% following rh-CD47p pretreatment, reaching virtually the same level of relaxation as the vehicle control at 92.0 ± 1.4% (Figure 4D).

### 3.5. Post-Treatment with rh-CD47p Completely Restored Vasodilation Following mTSP1-Mediated Injury to Endothelial Vasoreactivity

**Figure 5:**
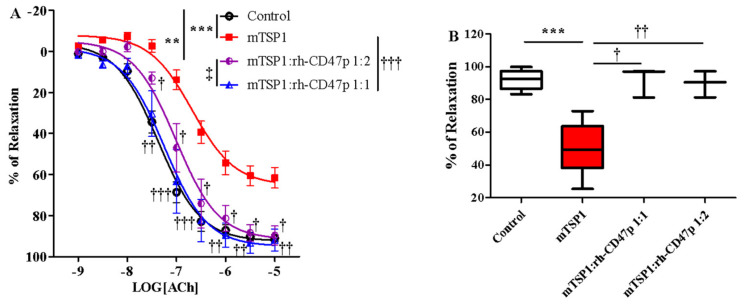
Post-treatment with recombinant human CD47 peptide (rh-CD47p) exerts restorative effect on injury to vasodilation by monomeric TSP1 (mTSP1). mTSP1 was incubated 15 min prior to coincubation with rh-CD47p for 45 min. (**A**) Concentration-response curves of isolated mouse thoracic aorta exposed to mTSP1 alone or the combination of mTSP1 and rh-CD47p in two different molar ratios. Substantial injury to vasodilation caused by mTSP1 was restored to control level (two-way ANOVA followed by a Bonferroni posthoc test; *n* = 4, *** *p* < 0.001, mTSP1 vs. control; ** *p* < 0.01, mTSP1:rh-CD47p 1:2 vs. control; † *p* < 0.05, †† *p* < 0.01, and ††† *p* < 0.0001, mTSP1:rh-CD47p 1:1 or 1:2 vs. mTSP1; ‡ *p* < 0.05, mTSP1:rh-CD47p 1:1 vs. 1:2). (**B**) Percentages of relaxation at 10 μM acetylcholine are presented as box and whisker plots showing median, 1st quartile, 3rd quartile, and 95% CI. Two doses of rh-CD47p brought impaired vasorelaxation close to control (*n* = 4, *** *p* < 0.0001, TSP1 vs. control; † *p* < 0.05, mTSP1:rh-CD47p 1:1 vs. mTSP1; †† *p* < 0.01, mTSP1:rh-CD47p 1:2 vs. mTSP1).

Pharmacological effectiveness of rh-CD47p as a therapeutic means to offset TSP1-impaired vasodilation was initially examined under an experimental post-treatment setting, utilizing monomeric TSP1 as a functional low-molecular-weight template for the large physiological trimeric form of TSP1. Two molar doses of DRP, in 1:1 and 1:2 molar ratios of mTSP1 to rh-CD47p, were evaluated, generally representing a low and a medium dosage level, respectively. Both tested DRP molar doses significantly improved vasodilation, noted with a minor difference in efficacy (Figure 5A, *p* < 0.05, mTSP1:rh-CD47p 1:1 vs. mTSP1:rh-CD47p 1:2). An equimolar dose of rh-CD47p to mTSP1 successfully restored mTSP1-inhibited vasodilation to vehicle control levels, and doubling the molar ratio of rh-CD47p to mTSP1 also significantly recovered mTSP1-inhibited vasodilation, just shy of reaching the vehicle control level (Figure 5A, *p* < 0.01, mTSP1:rh-CD47p 1:2 vs. control). Overall, both selected rh-CD47p dose levels steadily reestablished responsiveness to acetylcholine across its entire 1000-fold concentration range, following mTSP1 exposure equivalent to pathological levels of native TSP1 (Figure 5A, *p* < 0.01, <0.001, mTSP1:rh-CD47p 1:1 vs. mTSP1; *p* < 0.05, mTSP1:rh-CD47p 1:2 vs. mTSP1, respectively). Hence, a slightly greater improvement in the potency of acetylcholine was calculated for the treatment with a 1:1 dose (Table 1). Nevertheless, post-treatment with DRP at both molar ratio doses of 1:1 and 1:2 restored almost equal maximal levels of mTSP1-blunted vasorelaxation at 10 μM of acetylcholine, from 61.6 ± 5.0% back to 91.9 ± 5.3% and 89.7 ± 4.6%, respectively (Figure 5B).

### 3.6. Post-Treatment With Increasing Molar Ratios of rh-CD47p:tTSP1 to Achieve Complete Mitigation of tTSP1-Blunted Vasodilation 

**Figure 6:**
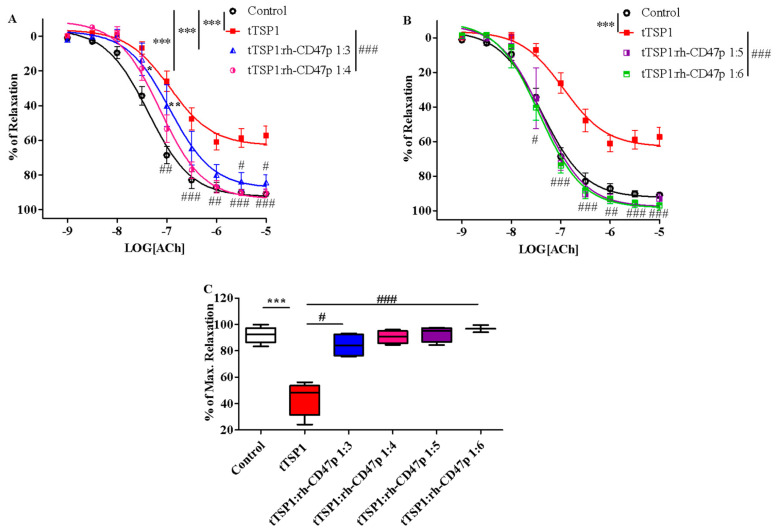
Post-treatment with recombinant human CD47 peptide (rh-CD47p) in low and high molar ratios differentially restored trimeric TSP1 (tTSP1)-blunted vasodilation. Acute exposure to tTSP1 for an initial 15 min was followed by 45 min coincubation with rh-CD47p. (**A**) Concentration-response curves of isolated mouse thoracic aorta exposed to tTSP1 alone or the combination of tTSP1 and rh-CD47p in low molar ratios (tTSP1:rh-CD47p 1:3 and 1:4). Low doses of rh-CD47p post-treatment ameliorated tTSP1-impaired vasodilation with limited efficacy (two-way ANOVA followed by a Bonferroni posthoc test; *n* = 3, * *p* < 0.05, ** *p* < 0.01, *** *p* < 0.001, tTSP1 alone, tTSP1:rh-CD47p 1:3 or 1:4 vs. control; # *p* < 0.05, ## *p* < 0.01, ### *p* < 0.001, tTSP1:rh-CD47p 1:3 or 1:4 vs. tTSP1). (**B**) Concentration-response curves of isolated mouse thoracic aorta exposed to tTSP1 alone or the combination of tTSP1 and rh-CD47p in high molar ratios (tTSP1:rh-CD47p 1:5 and 1:6). A total reversion of tTSP1-injured vasodilation was consistently observed in rh-CD47p post-treatment in high molar ratios (two-way ANOVA followed by a Bonferroni posthoc test; *n* = 4, *** *p* < 0.001, tTSP1 vs. control; # *p* < 0.05, ## *p* < 0.01, and ### *p* < 0.001, tTSP1:rh-CD47p 1:5 or 1:6 vs. tTSP1). (**C**) Percentages of maximal relaxation in response to 10 μM acetylcholine are presented as box and whisker plots showing median, 1st quartile, 3rd quartile, and 95% CI. Significantly reduced vasorelaxation observed in tTSP1-treated alone no longer existed after receiving low or high doses of rh-CD47p treatment (*n* = 3–4, *** *p* < 0.001, tTSP1 vs. control; # *p* < 0.05, tTSP1:rh-CD47p 1:3 vs. tTSP1; ### *p* < 0.001, tTSP1:rh-CD47p 1:4 up to 1:6 vs. tTSP1).

Following positive results against template mTSP1, rh-CD47p—as a therapeutic DRP design to antagonize TSP1-initiated detrimental effects—was further investigated under experimental pathological PAH-mimicking conditions (PAH is typically marked by pre-existent excess of TSP1 in circulation) [39]. This therapeutic scenario was created by exposing isolated aortic vessels to tTSP1 for 15 min [11], followed by titrated dosages of rh-CD47p treatment in 1:3 to 1:6 molar ratios of TSP1:DRP. Overall, all tested post-treatment DRP dosage ratios successfully counteracted TSP1-inhibited vasodilation, showing dose-related curative efficacies (Figure 6). The apparent nonlinear dose-dependent treatment effect of DRP was subsequently categorized into two general dose levels, i.e., a low molar dosage level, represented by 1:3 and 1:4 molar ratios, and a high dosage level, represented by 1:5 and 1:6 molar ratios. Both low-level dose ratios of DRP showed similar partial recovery of vasodilation following the TSP1 challenge (Figure 6A). The initial dose of the 1:3 molar ratio produced a modest yet significant recovery in vasodilation from tTSP1-treated alone and exhibited significantly improved vasorelaxation only at high concentrations of acetylcholine at 3 and 10 µM (*p* < 0.05, tTSP1:rh-CD47p 1:3 vs. tTSP1). Increasing the rh-CD47p dose to a 1:4 molar ratio improved vasodilation moderately by generating greater vasorelaxation across a wide range of acetylcholine, from 100 nM to 10 µM (*p* < 0.01, <0.001, tTSP1:rh-CD47p 1:4 vs. tTSP1). The dose of 1:4 also brought the sensitivity to acetylcholine closer to vehicle control compared to the dose of 1:3 (Table 1), demonstrating greater response to low concentrations of acetylcholine at 30 and 100 nM (*p* < 0.05, <0.01, tTSP1:rh-CD47p 1:3 vs. vehicle control).

Nevertheless, robust amelioration of tTSP1-mediated impairment of vascular tone was achieved at both high DRP molar dose ratios, 1:5 and 1:6, where vasorelaxations were fully restored to the same level as vehicle controls (Figure 6B). Such recovery of vasorelaxation encompassed a broader concentration range of acetylcholine, from as low as 30 nM up to 10 µM, produced by both of high doses of rh-CD47p (*p* < 0.05, <0.01, <0.001, tTSP1:rh-CD47p dose ratios of 1:5 and 1:6 vs. tTSP1). Consequently, vessels treated with either of the high doses of rh-CD47p were completely resistant to tTSP1-mediated suppression of sensitivity to vasorelaxant acetylcholine, demonstrating an almost identical vascular tone to that produced by the vehicle only control (Table 1). The lack of any additional vasorelaxation over that induced by the 1:5 dose ratio of TSP1:DRP generally indicates a plateau level of rh-CD47p antagonism against the tested pathological level of TSP1. The maximal vascular response achieved at the highest concentration of acetylcholine (10 μM) further confirmed the dose-dependent curative efficacies of DRP in our therapeutic model. Specifically, the low 1:3 molar dose of rh-CD47p considerably restored maximal vasorelaxation from 48.6 ± 6.8% in tTSP1 alone back to 84.2 ± 4.3%. Nonetheless, markedly higher maximum vasorelaxation recoveries, all the way back to the same level as the vehicle-only control, were achieved at 90.5 ± 2.4%, 93.1 ± 3.0%, and 96.9 ± 1.3%, following treatments with rh-CD47p doses of 1:4, 1:5, and 1:6, respectively (Figure 6C).

Altogether, rh-CD47p has shown several efficacy profiles, largely ameliorating pre-existing pathological TSP1-induced damage to vasorelaxation, whether in native or recombinant format.

## 4. Discussion

Elevated plasma or serum TSP1 levels in subjects with pre-existing conditions have been frequently reported and recognized as a biomarker for certain clinical diagnoses or prognoses. For instance, plasma TSP1 in patients with pulmonary hypertension can be as high as 1114 ± 136 ng/mL compared to controls at 82 ± 16 ng/mL [39] and 571 ± 226 ng/mL in acute ischemic stroke (AIS) versus 146 ± 50 ng/mL in control [40]. High expression of plasma TSP1 in AIS secondary to large vessel occlusion is correlated with diagnosis and prognosis with good or poor collaterals, reconciled by phase 1 of the SpecTRA clinical trial, revealing an increased odds ratio of TSP1 in minor stroke and transient ischemic attack [41]. Additionally, a high level of TSP1 in serum or plasma was tightly associated with atrial fibrillation or the new onset of atrial fibrillation [42,43]. More than a diagnostic or prognostic biomarker for several clinical conditions, excessive TSP1 plays pathologic roles in hypertension, pulmonary arterial hypertension, heart failure, and ischemia-reperfusion injury as well. Interaction between the C-terminus of TSP1 and its cognate receptor CD47 is necessary for TSP1-mediated inhibition of NO bioavailability, impairment of response to NO, and soluble guanylyl cyclase activity [15,44]. Till now, therapies targeting pathological excess of circulating TSP1 remain uncharted and somewhat ignored, partially due to the complexity of its structure and the associated molecular interactions. This is the first report of its kind to propose the unique concept of rh-CD47p as a DRP to disrupt interactions between TSP1 and its network and established its pharmacological efficacy to ameliorate TSP1-damaged vascular tone.

Direct interaction between TSP1 and its cognate receptor CD47 was demonstrated experimentally and described via computational modeling. Our initial in silico modeling revealed two regions of interactions between the C-terminus of TSP1 and the N-domain of the model extracellular domain of CD47, as suggested before. These molecular docking models pointed out that the proposed DRP molecule, the soluble recombinant human CD47 peptide (rh-CD47p) containing a TSP1-binding sequence, can specifically interact with the TSP1 molecule. Thus, as active DRP, rh-CD47p can compete with endogenous CD47 protein expressed on endothelial cell membranes to intercept the pairing of plasma TSP1 and endothelial CD47. Recombinant human CD47 peptide was previously reported as a research tool for experimental protein–protein interaction assays and modifying therapeutic delivery platforms [45,46]. This study is the first attempt to advance its application by exploiting the pharmacological characteristics of rh-CD47p. Qualitative and quantitative analyses of the simulated association between target protein TSP1 and the DRP molecule, rh-CD47p, were carried out in an exclusive in vitro setting. The apparent upward band shift of about 60 KDa in the mixture of mTSP1 and rh-CD47p from the single TSP1 control band was indicative of a direct association between both proteins. This was reaffirmed via the minimum band migration (from top of gel) observed when the TSP1 blocking antibody was simultaneously included in the protein mixture. As the immunoblot also clearly demonstrated, the TSP1 blocking antibody had no cross-reactivity with rh-CD47p, confirming the specific binding of TSP1 molecules to the DRP moiety, as proposed.

Stoichiometry between the target protein and DRP molecules was also examined utilizing modified ELISA for both the recombinant monomeric form and native TSP1 molecules. Regardless of the different observed TSP1-bound rates in mixtures with recombinant or native TSP1 (determined based on slopes in Figure 2C vs. Figure 2E), a strong linear DRP dose-dependent correlation was consistently calculated for both versions of the TSP1 protein. This finding not only substantiated that rh-CD47p as a DRP molecule is capable of binding to our target protein TSP1, independently of its format, but also provided a working dynamic dose range of the candidate DRP to further investigate its pharmacological effectiveness.

mTSP1 proved itself a useful tool in the binding assays. Still, it was not determined whether it can function as a smaller active pharmacological alternative for native tTSP1 until it exhibited a closely similar efficacy profile to that of its endogenous trimeric counterpart in the inhibition of endothelium-dependent vasodilation. Whether rh-CD47p is able to serve as a decoy receptor to block TSP1-mediated pathological effects by competing with the endogenous CD47 expressed on cell surfaces was pharmacologically investigated under two therapeutic scenarios: as a prophylactic agent against rising serum TSP1 levels (applicable in early diagnosed conditions) and as a treatment for pathologically elevated TSP1 levels (applicable in pre-existing conditions).

First, the candidate DRP was assessed for prophylaxis against TSP1-enhanced vasoconstriction using an ex vivo model of isolated mouse thoracic aorta. rh-CD47p successfully demonstrated its decoy receptor activity, as proposed, to circumvent a TSP1/CD47 axis-initiated signaling cascade and the loss of endothelial vascular tone, thus allowing the recovery of vasodilation to basal levels (commonly through modulation of NO production and NO-initiated vascular activities). Such promising ex vivo results encouraged further evaluation of the therapeutic effect of the proposed DRP to antagonize excessive vasoconstriction induced by pre-existing excess TSP1, generally reported in multiple clinical conditions. It is known that various vascular activities of NO occur over different temporal scales, from seconds to days [12,37]; then, it is reasonable that TSP1 modulates NO-related events in different time frames. For instance, TSP1 suppresses endothelium-derived NO production by inhibiting eNOS activity in as short as 15 min [11] and reduces NO bioavailability by inducing superoxide generation by vascular smooth muscle cells in about an hour [13]. Accordingly, the therapeutic effects of DRP were evaluated in the post-treatment study scenario following acute exposure to TSP1 for 15 min, mimicking the sudden pathological surge of TSP1 released from platelet α-granules during rapid activation. At different dose levels, post-treatment with rh-CD47p further affirmed the ameliorating potential of DRP against pathological TSP1-mediated vasodilation impairment, which was also recorded for both native and recombinant TSP1 protein isoforms. Conceptually, DRP offers immunity to TSP1 impairment of vasorelaxation, predominately via competing with endogenous surface CD47 for binding sites at TSP1, thus circumventing the direct binding of TSP1 to its endothelial cell receptor, CD47, and all subsequent intravascular molecular interactions.

Determining the effective dose range of DRP vs. target protein TSP1 is of fundamental importance for the pharmaceutical development of DRP-based medicine prototypes for subsequent preclinical evaluation against specific animal disease models. In order to examine the effectiveness of rh-CD47p against high levels of TSP1 in clinical conditions, the concentration of native tTSP1 in the current report replicated the highest TSP1 pathological level detected in PAH patients, for example [39]. Analogously, the selected concentration of the recombinant human mTSP1 template also reproduced marked inhibition of endothelium-dependent vasorelaxation comparable to that of the native form. Based on dose titration in quantitative binding assays, tTSP1:rh-CD47p 1:3 and mTSP:rh-CD47p 1:1 were rationally identified as starting test doses in both pre- and post-treatment protocols for the assessment of prophylactic and therapeutic efficacies, respectively. The dose of tTSP1:rh-CD47p 1:3 was enough to prevent interaction between tTSP1 and endothelial CD47, whereas a molar ratio dose of tTSP1:rh-CD47 at 1:5 or higher was found necessary to completely restore endothelium-derived NO reactivity in the presence of pre-existing tTSP1. At the same time, with mTSP1, a close higher dose ratio of mTSP1:rh-CD47p 1:2 was seemingly needed to achieve full prophylaxis. On the other hand, post-treatment with this dose largely renovated endothelium-dependent vasorelaxation almost back to the control levels, while the next lower dose of mTSP1:rh-CD47p 1:1 mostly resulted in a pronounced recovery in vascular tone. The slight difference in responses under the same dose between pre- versus post-treatment protocols can be explained by variations in pharmacological reactivity of the TSP1 peptide. Altogether, the minimum effective DRP molar dose was thus determined to be at 1:3 of tTSP1:rh-CD47p or at 1:1 of mTSP1:rh-CD47p based on our conclusive ex vivo model data. Thus far, the proof of concept of rh-CD47p as a DRP to circumvent circulatory TSP1 and endothelial CD47 interaction has been demonstrated, along with an effective dose range to counteract TSP1-mediated vascular tone impairments. These strong findings establish the basic foundation to support further development of a pharmaceutical prototype of DRP against TSP1, starting with the identified minimum effective dosages in each proposed therapeutic scenario.

**Figure 7:**
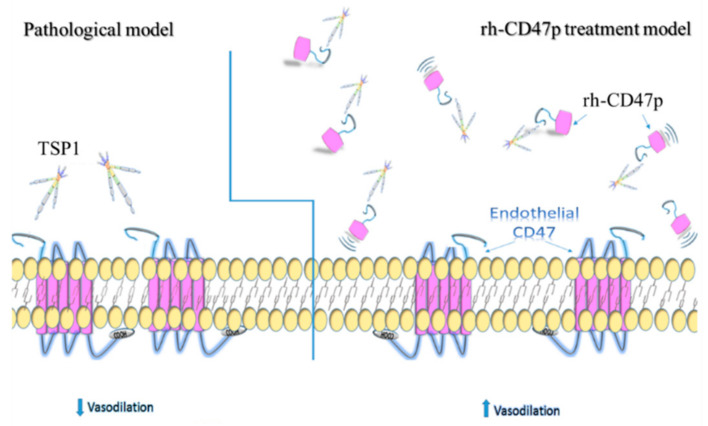
Schematics illustrating the putative alleviation mechanism of TSP1-impaired vasodilation via administration of recombinant human CD47 peptide (rh-CD47p). Under pathological conditions, excessive TSP1 is present in circulation or acutely excreted upon damage to the vascular wall. Under either pathological condition, excessive TSP1 significantly alters vascular tone via binding to its cognate receptor CD47 expressed on vascular cells, presenting as inhibited endothelium-dependent vasodilation. rh-CD47p, containing the TSP1 binding domain, was given prior to or after exposure to TSP1 to interfere with the communication between TSP1 and endothelial CD47, showed in the panel of the rh-CD47p treatment model. Since the signaling cascade initiated upon the interaction between TSP1 and endothelial CD47, TSP1-impaired endothelium-dependent vasodilation was expected to be prevented or restored as rh-CD47p was administered prior to or after the TSP1 challenge, respectively.

The pivotal role of elevated TSP1 in the pathogenesis of occlusive diseases, such as pulmonary arterial hypertension, is not limited to promoting vasoconstriction, which is only tested in this report. Vascular remodeling secondary to long-term pathological TSP1 exposure, including migration and proliferation of pulmonary vascular smooth muscle cells and fibroblasts, precipitates medial thickening and fibrosis. TSP1-upregulated TGFβ signaling was also revealed to be heavily involved in the progression of fibrosis in both vascular and pulmonary tissue [47,48]. Collectively, our data support the proposed active DRP design, rh-CD47p, as a potential strategy to abrogate TSP1-mediated endothelial impairment and subsequent vascular damage, regardless of pre-existing or a sudden surge of TSP1 levels. It is also important to note that while mechanisms regulating the vasoreactivity in conduit vessels versus resistance vessels are different, reports have revealed that TSP1/CD47 signaling leads to resistance vascular dysfunction and vasculopathy (e.g., in pulmonary arteries and femoral resistance arteries) in the same manner as systemic vasculature, i.e., by hampering vasodilation and promoting vasoconstriction [8,13]. Further pharmacological investigation of this anti-TSP1 rh-CD47p strategy, to be conducted in resistance vessel models, would help delineate the effective dosing profiles. A pharmaceutical DRP therapy against TSP1, as illustrated in Figure 7, can likely help to combat the detrimental tissue remodeling effects of TSP1 in addition to normalizing excessive vasoconstriction, as demonstrated in these studies. Our proof-of-principle report strongly supports further investigation of rh-CD47p-based therapy in different preclinical pathological models that implicate elevated TSP1. Based on such promising early-stage pharmacological data, additional single- and multiple-dose evaluations of our DRP-therapy in diseased animal models, such as ischemic reperfusion injury or pulmonary arterial hypertension, would be warranted. In vivo pharmacokinetic analysis to determine the plasma half-life of parenteral rh-CD47p-based therapy and the rh-CD47p-TSP1 elimination complex would serve as a prerequisite to establishing the general dosing parameters for all subsequent preclinical and clinical studies. This current proof-of-concept report opens up prospects to explore the broad potential pharmacotherapeutic applications of our TSP1-targeting rh-CD47p-based therapy through the development of advanced pharmaceutical platforms. One possibility would be rh-CD47p as a fusion peptide, to be constructed with the Fc portion of the human IgG antibody. Another can be as nanocarriers coated with multiple active DRP molecules, designed for systemic administration and capable of scavenging and eliminating pathological TSP1-plasma levels, which are generally implicated in acute clinical conditions such as cerebrovascular or myocardial IRI and PAH.

## Figures and Tables

**Table 1:** Potency of acetylcholine, measured in EC_50_ (mean ± SEM, nM), in recombinant human CD47 peptide (rh-CD47p) pretreatment and post-treatment cohorts.

Cohort	EC_50_	Fold of Shift vs. Control	Fold of Shift vs. TSP1 Alone
Control	43.5 ± 6.3		
tTSP1 alone	213.8 ± 81.5 *	4.9	
mTSP1 alone	238.6 ± 76.7 ^†^	5.5	
Pre-treatment			
tTSP1:CD47 1:3	59.7 ± 9.1 *	1.4	−3.6
mTSP1:CD47 1:1	130.2 ± 31.1 ^†^	3.0	−1.8
mTSP1:CD47 1:2	47.8 ± 3.2 ^†^	1.1	−5.0
Post-treatment			
tTSP1:CD47 1:3	147.9 ± 48.7	3.4	−1.4
tTSP1:CD47 1:4	79.8 ± 17.9 ^##^	1.8	−2.7
tTSP1:CD47 1:5	45.3 ± 17.0 ^##^	1.0	−4.7
tTSP1:CD47 1:6	44.4 ± 8.0 ^##^	1.0	−4.8
mTSP1:CD47 1:1	74.8 ± 32.1 ^†^	1.9	−3.2
mTSP1:CD47 1:2	172.1 ± 68.5 ^†^	4.0	−1.4

Trimeric TSP1 (tTSP1); Monomeric TSP1 (mTSP1); Half maximal effective concentration: EC_50_. Two-tailed Student’s *t*-test. *, *p* < 0.05, compared to control; †, *p* < 0.05, compared to mTSP1 control; ##, *p* < 0.01, compared to tTSP1 control. −, indicates leftward shift to higher potency.

## Data Availability

The data presented in this study are available on request from the corresponding authors.
